# The Short-Chain Fatty Acid Uptake Fluxes by Mice on a Guar Gum Supplemented Diet Associate with Amelioration of Major Biomarkers of the Metabolic Syndrome

**DOI:** 10.1371/journal.pone.0107392

**Published:** 2014-09-09

**Authors:** Gijs den Besten, Rick Havinga, Aycha Bleeker, Shodhan Rao, Albert Gerding, Karen van Eunen, Albert K. Groen, Dirk-Jan Reijngoud, Barbara M. Bakker

**Affiliations:** 1 Center for Liver, Digestive and Metabolic Diseases, Department of Pediatrics & Systems Biology Center for Energy Metabolism and Ageing, University of Groningen, University Medical Center Groningen, Groningen, The Netherlands; 2 Department of Laboratory Medicine, University of Groningen, University Medical Center Groningen, Groningen, The Netherlands; 3 Netherlands Consortium for Systems Biology, Amsterdam, The Netherlands; 4 Top Institute Food and Nutrition, Wageningen, The Netherlands; Southern Illinois University School of Medicine, United States of America

## Abstract

Studies with dietary supplementation of various types of fibers have shown beneficial effects on symptoms of the metabolic syndrome. Short-chain fatty acids (SCFAs), the main products of intestinal bacterial fermentation of dietary fiber, have been suggested to play a key role. Whether the concentration of SCFAs or their metabolism drives these beneficial effects is not yet clear. In this study we investigated the SCFA concentrations and *in vivo* host uptake fluxes in the absence or presence of the dietary fiber guar gum. C57Bl/6J mice were fed a high-fat diet supplemented with 0%, 5%, 7.5% or 10% of the fiber guar gum. To determine the effect on SCFA metabolism, ^13^C-labeled acetate, propionate or butyrate were infused into the cecum of mice for 6 h and the isotopic enrichment of cecal SCFAs was measured. The *in vivo* production, uptake and bacterial interconversion of acetate, propionate and butyrate were calculated by combining the data from the three infusion experiments in a single steady-state isotope model. Guar gum treatment decreased markers of the metabolic syndrome (body weight, adipose weight, triglycerides, glucose and insulin levels and HOMA-IR) in a dose-dependent manner. In addition, hepatic mRNA expression of genes involved in gluconeogenesis and fatty acid synthesis decreased dose-dependently by guar gum treatment. Cecal SCFA concentrations were increased compared to the control group, but no differences were observed between the different guar gum doses. Thus, no significant correlation was found between cecal SCFA concentrations and metabolic markers. In contrast, *in vivo* SCFA uptake fluxes by the host correlated linearly with metabolic markers. We argue that *in vivo* SCFA fluxes, and not concentrations, govern the protection from the metabolic syndrome by dietary fibers.

## Introduction

The shift in diet in Western and developing countries from a traditional high-fiber, low-fat, low-calorie diet towards a low-fiber, high-fat, high-calorie diet is accompanied with a growing prevalence of the metabolic syndrome comorbidities: obesity, hypertension, dyslipidemia and insulin resistance [1], [2]. Epidemiological and large observational studies reported an inverse correlation between dietary fiber intake and body weight, insulin resistance, hypertension and dyslipidemia [3]. The dietary fiber guar gum is especially promising, as it has been shown to decrease hypercholesterolemia, hyperglycemia and obesity in multiple experiments in both rodents and humans [4], [5]. The molecular mechanisms by which guar gum induces these effects constitute an active field of research. Short-chain fatty acids (SCFAs), the main products of intestinal bacterial fermentation of dietary fiber, have been suggested to play a key role in these beneficial effects [6].

SCFAs are saturated aliphatic organic acids that consist of 1–6 carbons of which acetate (C2), propionate (C3) and butyrate (C4) are the most abundant (≥95%) [7]. In the last decades it became apparent that SCFAs might play a key role in the prevention and treatment of the metabolic syndrome, bowel disorders and certain types of cancer [8]–[14]. The effects of dietary fiber on the host are mostly studied by looking at the fecal or colonic SCFA concentrations and the host physiology. However, increased concentrations of SCFAs do not always correlate to beneficial host effects. For instance, genetically obese *ob/ob* mice and obese human subjects had increased concentrations of cecal and fecal SCFAs as compared to lean controls [15]–[17], while germ-free mice and rats had low SCFA concentrations and were protected from diet-induced obesity [18], [19]. Recently, Teixeira *et al*. [20] even suggested that human fecal SCFA concentrations in women are *positively* correlated with metabolic syndrome risk factors such as adiposity, waist circumference and HOMA index. These results raise the question if SCFAs are involved in the beneficial effect of dietary fibers. It is important to note, however, that at steady-state the cecal or fecal SCFA concentrations not necessarily reflect the SCFA uptake fluxes by the host. It is known that SCFAs exert their effects not only directly in the gut, but also via other organs like the liver and adipose tissue [6]. For the latter effect the cecal concentration is of less importance than the amount of SCFAs that is transported into the host. It is plausible that the SCFA host uptake fluxes are involved in the beneficial effect of dietary fibers. *In vivo* flux measurements, however, are challenging. Therefore, SCFA production fluxes have been measured mostly *in vitro* by exposing an inoculum of gut microbiota to dietary fiber. The disadvantages of this method are that *i*) during isolation of the anaerobic gut microbiota the diversity decreases, *ii*) raw substrates are not modified as it normally occurs *in vivo* in the upper part of the gastrointestinal tract, *iii*) products accumulate during fermentation due to the lack of host uptake mechanisms and *iv*) the uptake fluxes by the host, which we ultimately need to know, cannot be determined [21], [22].

In this study we present a novel method to determine *in vivo* SCFA fluxes under different dietary conditions. The method was based on infusion of tracer amounts of ^13^C-labeled acetate, propionate or butyrate into the cecum and the results of all three infusions were combined in a single steady-state isotope model. This allowed us to quantify how the intake of 0%, 5%, 7.5% or 10% of the dietary fiber guar gum affected the SCFA fluxes and how these correlated with major biomarkers of the metabolic syndrome.

## Materials and Methods

### Ethics Statement

The national and institutional guidelines for the care and use of animals were followed, and the experimental procedures were reviewed and approved by the Ethics Committees for Animal Experiments of the University of Groningen, The Netherlands (ethics registration code 5887). All efforts were made to minimize suffering.

### Animals and Experimental Design

Male C57Bl/6J mice (Charles River, L'Arbresle Cedex, France), 2 months of age, were housed in a light- and temperature-controlled facility (lights on 6:30 a.m. to 6:30 p.m., 21°C). During 6 weeks mice were fed a high-fat semi-synthetic diet (based on D12451 [23], Research Diet Services, Wijk Bij Duurstede, The Netherlands) in which 0, 5, 7.5 or 10% (w/w) guar gum (Viscogum MP 41230, Cargill, United States) replaced an equivalent amount of corn starch. In this way we ensured that only the fiber content was varied while the total polysaccharide and calorie content of the diets remained equal. Mice had free access to food and drinking water. The Ethics Committee for Animal Experiments of the University of Groningen approved the experimental procedures.

### Plasma and tissue sampling

Mice were fasted from 8–12 a.m. Blood glucose concentrations were measured with a EuroFlash meter (Lifescan Benelux, Beerse, Belgium). Blood samples were drawn by tail bleeding into heparinized tubes. Blood was centrifuged (4000×*g* for 10 min at 4°C) and plasma was stored at −20°C. Plasma insulin levels were determined using ELISA (ALPCO Diagnostics, Salem, United States) and HOMA-IR was calculated (IR  =  (fasting insulin in mU/L x fasting glucose in mM)/22.5). Plasma triglycerides concentrations were determined using a commercially available kit (Roche). Hepatic TG content was determined after lipid extraction [24].

### Hyperinsulinemic-euglycemic clamps

Mice were operated for jugular vein catherization after 6 weeks on a 0% or 10% guar gum diet and insulin sensitivity was determined as previously described [25]. In brief, mice were fasted for 9 h and were infused for a 3 h basal period with an isotonic saline solution containing [U-13C] glucose (7 mM), (Cambridge Isotope Laboratories, Andover, MA) at an infusion rate of 0.54 ml/h. For the hyperinsulinemic condition, solutions were changed after 3 h, and mice were subjected to a 3 h hyperinsulinemic period by infusing an isotonic saline solution containing insulin (44 mU/ml, Actrapid; Novo Nordisk, Denmark), somatostatin (40 µg/ml; UCB, The Netherlands) and 1% BSA (Sigma, St. Louis, MO) at a constant rate of 0.135 ml/h. During hyperinsulinemia, euglycemia was kept by infusion of a second solution that contained 27% glucose (1.6 M) and 3% [U-13C]glucose (50 mM) at an adjustable rate to maintain plasma glucose levels. During the experiment, blood glucose levels were measured every 15 min in blood drops collected by tail tip bleeding. Every 30 min, blood spots for GC-MS analysis were taken by tail tip bleeding on filter paper, air dried, and stored at room temperature until further analysis. Urine samples were collected on filter paper at hourly intervals. Glucose fluxes were calculated using mass isotopologue distribution analysis (MIDA) as previously described [26].

### Cecal infusion experiment

After 6 weeks on diet, mice were equipped with a permanent cecum catheter and allowed a recovery period of at least 5 days as described previously [27]. Cecal cannulas were flushed daily with phosphate buffered saline. On the day of the experiment, mice were individually housed and fasted from 6:00 to 10:00 a.m. All infusion experiments were performed in conscious, unrestrained mice. For each dietary treatment group, three different groups received solutions of phosphate buffered saline containing either sodium [1-^13^C]acetate (3 mM, 99 atom %, Sigma-Aldrich), sodium [2-^13^C]propionate (1.5 mM, 99 atom %, Sigma-Aldrich) or sodium [2,4-^13^C_2_]butyrate (0.6 mM, 99 atom %, Sigma-Aldrich) via the cecum catheter at an infusion rate of 0.2 ml/h. After 6 h of infusion, animals were sacrificed by cardiac puncture under isoflurane anesthesia. Cecum content was removed quickly, frozen in liquid nitrogen, and stored at -80°C for SCFA enrichment determination.

### Determination of SCFA concentrations and enrichments

Cecal concentrations and enrichments of SCFAs were measured as previously described [27]. In short, cecum content was centrifuged and 25 µl of supernatant was spiked with 25 µl of internal standard (17.3 mM hydroxyisocapronic acid) and 5 µl of 20% 5-sulfosalicyclic acid. After a 10 min centrifugation the supernatant was acidified with 2.5 µl 37% HCl and SCFA were extracted with 2 ml diethylether. Derivatization was performed overnight with 500 µl supernatant and 50 µl of *N*-*tert*-Butyldimethylsilyl-*N*-methyltrifluoroacetamide (MTBSTFA).

Mass isotopologue distributions were measured in an Agilent 5975 series GC/MSD (Agilent Technologies). The gas chromatograph was equipped with a ZB-1 column (Phenomenex). Mass spectrometry analysis was performed by electron capture negative ionization with methane as the moderating gas. Ions monitored were ^m^/_z_ 117–118 for acetate, ^m^/_z_ 131–132 for propionate and ^m^/_z_ 145–147 for butyrate. The normalized mass isotopologue distributions measured by GC-MS (m_0_-m_x_) were corrected for natural abundance of ^13^C by multiple linear regression according to Lee *et al*. [28] to obtain the excess fractional distribution of mass isotopologues (M_0_-M_x_).

### Gene expression levels

RNA was extracted from cecum tissue using Tri reagent (Sigma-Aldrich, St. Louis, MO) and converted into cDNA by a reverse transcription procedure using M-MLV and random primers according to the manufacturer's protocol (Sigma-Aldrich). For quantitative PCR (qPCR), cDNA was amplified using the appropriate primers and probes. Taqman RT-PCR primer and probe were used to determine mRNA for MCT-1 (Mm01315398_m1), SMCT-1 (Mm00520629_m1), PEPCK (Mm01247058_m1), G6Pase (Mm00839363_m1), PC (Mm00500992_m1), HK (Mm00439344_m1), PK (Mm00443090_m1), FASN (Mm01204974_m1), ACC1 (Mm01304257_m1), ACC2 (Mm01204671_m1) and ELOVL6 (Mm00851223_s1). mRNA levels were calculated relative to 36b4 (Mm00725448_s1) expression and normalized for expression levels of mice fed the control diet.

### Statistics

All data are presented as mean values ± SEM. Statistical analysis was assessed by one-way ANOVA using the Tukey test for post-hoc analysis. For analysis of correlations, Spearman's rank test was used. Statistical significance was defined as a p value below 0.05. Data were analyzed with SPSS v.20 software.

## Results

### Guar gum protects against diet-induced obesity and insulin resistance in a dose-dependent manner

**Figure 1:**
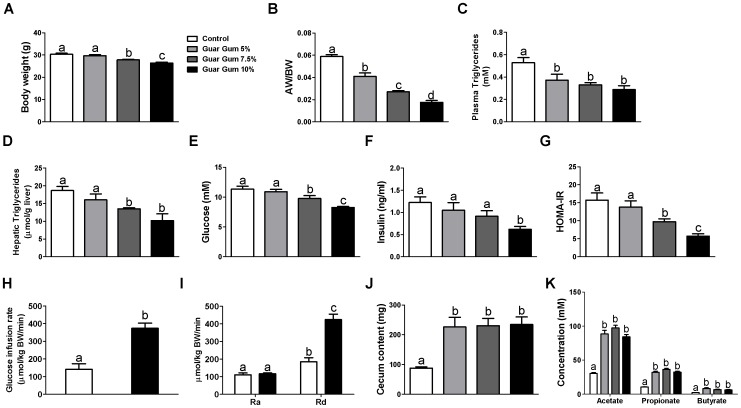
Effect of dietary supplementation with guar gum on mouse physiology after 6 weeks on a high-fat diet. (A) Body weight for the different guar gum groups. (B) Adipose weight body weight ratio (AW/BW) for the different guar gum groups. Triglycerides in plasma (C) and liver (D). Plasma glucose (E) and insulin (F) levels after a 4-hour fast. Blood glucose and insulin levels were used to determine insulin sensitivity through HOMA-IR (G). (H) Average glucose infusion rates needed to maintain euglycemic conditions during hyperinsulinemic-euglycemic clamps (HIEC) conditions for the 0% and 10% guar gum groups. (I) Hepatic glucose production (Ra) and peripheral glucose disposal (Rd) rate during HIEC conditions. (J) Cecal content and (K) cecal SCFA concentrations. Data represent means ± SEM for n = 7–8. Different letters indicate significant differences between groups (at least p<0.05).

Supplementation of a high-fat diet with guar gum dose-dependently decreased body weight of mice after 6 weeks treatment (Figure 1A), with a maximal effect at the highest dose of the fiber (13% decrease at 10% guar gum vs. control; p<0.001). In concert, adipose/body weight ratio decreased dose-dependently by guar gum treatment (70% decrease at 10% guar gum vs. control; p<0.001; Figure 1B). Triglycerides in plasma and liver also decreased with an increasing dose of guar gum (46% decrease at 10% guar gum vs. control; p<0.001 and 45% decrease at 10% guar gum vs. control; p<0.01, respectively; Figure 1C and 1D). Expression of genes involved in hepatic fatty acid synthesis was decreased with increasing dosage of guar gum, while no effect was observed for genes involved in fatty acid oxidation (Table 1). Fasted plasma glucose and insulin levels decreased dose-dependently by guar gum treatment (27% decrease at 10% guar gum vs. control; p<0.001 and 49% decrease at 10% guar gum vs. control; p<0.01, respectively; Figure 1E and 1F). Expression of genes involved in hepatic gluconeogenesis was decreased with increasing dosage of guar gum, while expression of genes involved in glycolysis were increased in a guar gum dose-dependent manner (Table 1). The homeostasis model assessment for insulin resistance (HOMA-IR) decreased with an increasing dose of guar gum (64% decrease at 10% guar gum vs. control; p<0.001; Figure 1G). To quantify insulin sensitivity and to discriminate between the effects of guar gum on liver and peripheral insulin action, we performed hyperinsulinemic euglycemic clamp studies under matched insulin exposure for control and 10% guar gum fed mice. The glucose-infusion rate required for maintaining euglycemia (a measure of whole-body insulin sensitivity) in guar gum-fed mice was significantly higher than that in control mice (164% increase at 10% guar gum vs. control; p<0.001; Figure 1H). While the hepatic glucose production rate during the clamp was similar in both groups (Ra in Figure 1I), the degree to which insulin stimulated the rate of glucose uptake by peripheral tissues (primarily muscle and adipose tissue) was much higher in guar gum-fed mice (130% increase at 10% guar gum vs. control; p<0.001; Rd in Figure 1I). All groups of mice treated with guar gum showed an approximately 2.5-fold increase in cecal mass content compared to the control diet (167% increase at 10% guar gum vs. control; p<0.01; Figure 1J). However, in this respect no differences were observed between the different guar gum groups. Cecal concentrations of acetate (176% increase at 10% guar gum vs. control; p<0.001), propionate (207% increase at 10% guar gum vs. control; p<0.001) and butyrate (174% increase at 10% guar gum vs. control; p<0.05) were increased in the three guar gum groups compared to the control group (Figure 1K), but again no significant differences were observed between the different guar gum groups. The cecal mRNA expression of two known colonic SCFA transporters, monocarboxylate transporter 1 (MCT-1) and sodium-coupled MCT-1 (SMCT-1), were increased in the guar gum groups compared to the control group, with the highest expressions in the 5% guar gum group (Table 1).

**Table 1:** Gene expression for cecal SCFA transport, hepatic gluconeogenesis and glycolysis and hepatic fatty acid synthesis and oxidation for the different guar gum groups.

	Control	Guar Gum 5%	Guar Gum 7.5%	Guar Gum 10%
*SCFA transport*				
Mct-1	1.00±0.13^a^	1.62±0.06^b^	1.32±0.13^a^	1.23±0.06^a^
Smct-1	1.00±0.09^a^	4.55±0.48^b^	4.14±0.31^b^	3.08±0.13^c^
*Gluconeogenesis*				
Pepck	1.00±0.11^a^	0.90±0.11^a^	0.64±0.05^b^	0.50±0.08^c^
G6Pase	1.00±0.14^a^	0.70±0.10^b^	0.47±0.11^c^	0.33±0.03^c^
PC	1.00±0.11^a^	0.87±0.04^a^	0.79±0.07^b^	0.74±0.06^b^
*Glycolysis*				
HK	1.00±0.07^a^	1.50±0.19^a^	2.08±0.33^b^	3.02±0.53^c^
PK	1.00±0.10^a^	1.10±0.12^a^	1.45±0.14^b^	1.69±0.19^b^
*Fatty acid synthesis*				
Fasn	1.00±0.04^a^	0.71±0.06^b^	0.69±0.08^b^	0.51±0.05^c^
Acc1	1.00±0.08^a^	0.84±0.05^a^	0.73±0.08^b^	0.66±0.06^b^
Acc2	1.00±0.09^a^	0.88±0.10^a^	0.83±0.08^b^	0.56±0.05^b^
Elovl6	1.00±0.14^a^	0.86±0.07^a^	0.75±0.07^b^	0.67±0.08^b^
*Fatty acid oxidation*				
Cpt-1a	1.00±0.07^a^	0.88±0.11^a^	0.84±0.06^a^	0.84±0.07^a^
Mcad	1.00±0.06^a^	1.10±0.12^a^	1.01±0.09^a^	1.01±0.10^a^
Lcad	1.00±0.04^a^	0.94±0.08^a^	0.96±0.07^a^	0.94±0.07^a^
Aox	1.00±0.04^a^	1.03±0.04^a^	1.12±0.07^a^	0.96±0.06^a^

Mct-1, Monocarboxylate transporter 1; Smct-1, sodium-coupled monocarboxylate transporter 1; Pepck, phosphoenolpyruvate carboxykinase; G6Pase, glucose 6-phosphatase; PC, pyruvate carboxylase; HK, hexokinase; PK, pyruvate kinase; Fasn, fatty acid synthase; Acc1, acetyl-CoA carboxylase 1; Acc2, acetyl-CoA carboxylase 2; Elovl6, fatty acid elongase 6; Cpt-1a, carnitine palmitoyltransferase 1a; Mcad, medium-chain acyl coA dehydrogenase; Lcad, long-chain acyl coA dehydrogenase; Aox, acyl-CoA oxidase.

Data represent means ± SEM for n = 7–8. When groups have a different superscript *a*, *b* or *c* associated, the results differ significantly between them (at least p<0.05).

### Short-chain fatty acid concentrations correlate with cecal transporter mRNA expression but not with metabolic syndrome markers

**Figure 2:**
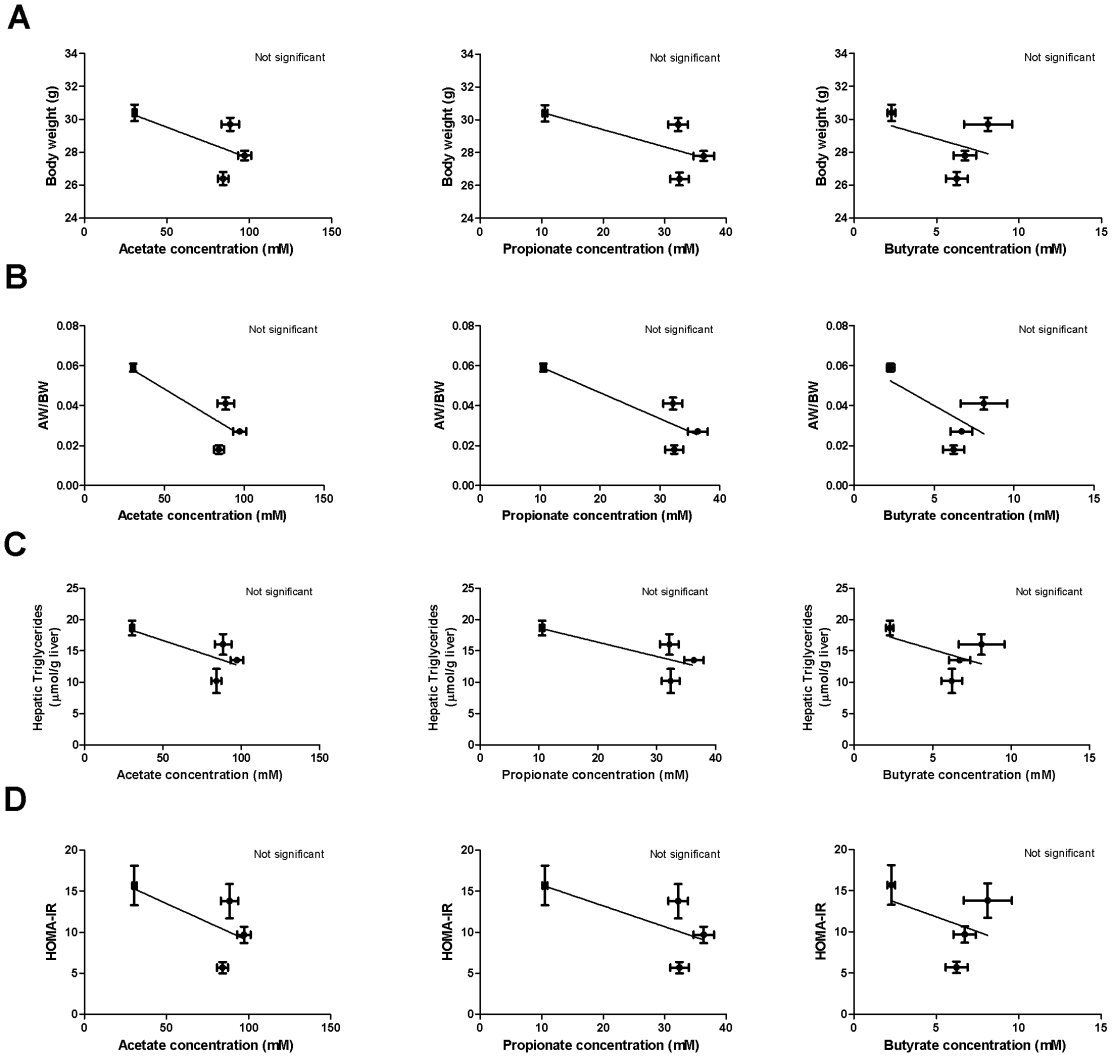
Cecal SCFA concentrations correlations. Correlation of cecal acetate, propionate and butyrate concentration with body weight (A), AW/BW (B), hepatic triglycerides (C) and HOMA-IR (D). The Spearman's correlation coefficient was calculated and the significance level was set at p<0.05.

To investigate if the concentration of cecal SCFAs associate with markers of the metabolic syndrome (*i.e*. body weight, adipose weight, triglycerides, glucose, insulin and HOMA-IR), we plotted the concentration of the three SCFA against the metabolic syndrome markers of the different treatment groups. Cecal acetate, propionate and butyrate concentrations did not correlate with body weight, adipose weight, triglycerides, glucose, insulin and HOMA-IR (p>0.05; Figures 2 and S1). In contrast, all three SCFAs correlated significantly with SMCT-1 and MCT-1 expression but not with genes involved in gluconeogenesis, glycolysis, fatty acid synthesis and fatty acid oxidation (Table S1). We hypothesized that the physiological effect of guar gum was not exerted via the colonic SCFA concentration, but rather via the SCFAs that are produced by the microbiota and taken up by the host. Therefore, we set out to determine these fluxes *in vivo*.

### A model to determine short-chain fatty acid production and uptake fluxes

**Figure 3:**
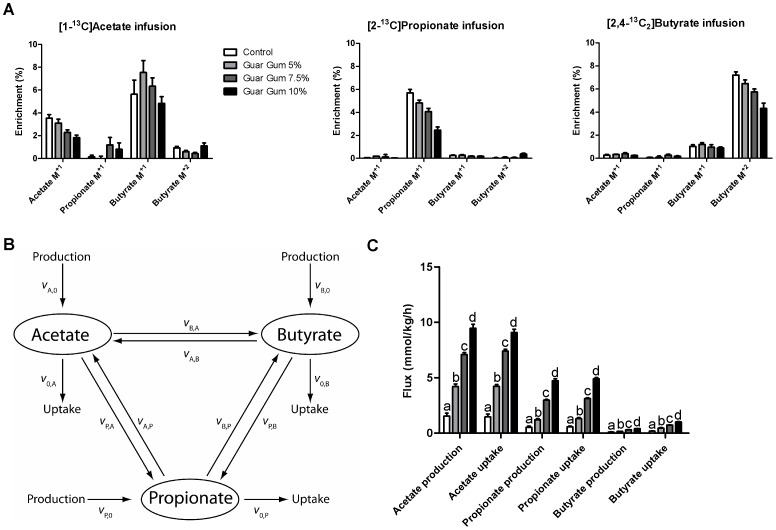
*In vivo* SCFA fluxes. (A) Enrichment of cecal SCFAs after 6 h infusion with [1-^13^C] acetate, [2-^13^C] propionate or [2,4-^13^C_2_] butyrate for the different guar gum groups. (B) Schematic overview of the model used to determine *in vivo* bacterial SCFA production, interconversion and host uptake fluxes at steady state. Each reaction is represented by a flux (*v*, for a detailed description see Text S1). (C) *In vivo* SCFA production and uptake fluxes for the different guar gum groups after 6 weeks on high-fat diet. Data represent means ± SEM for n = 7–8. Different letters indicate significant differences between groups (at least p<0.05).

We designed an animal experiment to determine *in vivo* fluxes of SCFA production, interconversion and uptake by the host, based on isotope dilution and incorporation. We infused separately tracer amounts of [1-^13^C]acetate, [2-^13^C]propionate or [2,4-^13^C_2_]butyrate during 6 h into the cecum of conscious, unrestrained mice and measured the label content in the cecal SCFAs at the end of the infusion period as described before [27]. Infusion of any of the labeled SCFAs resulted in label incorporation in all three cecal SCFAs (Figure 3A). This indicates that there is interconversion of labeled SCFAs by the gut bacteria. To calculate the *in vivo* fluxes of bacterial SCFA production and consumption by the host we constructed a mathematical model that accounts for this bacterial label conversion (Figure 3B). Starting from the assumption that the isotopic measurements were performed during mass and isotopic steady state, flux balance equations were derived for each of the labeled and unlabeled SCFAs during the tracer infusions of [1-^13^C] acetate, [2-^13^C] propionate or [2,4-^13^C_2_] butyrate. We further assumed that the labeled tracers did not affect the total mass fluxes and hence these should be identical in the three infusion experiments. Based on the observation that double-labeled butyrate was detected after infusion of acetate and propionate, we assumed that 2 acetate or propionate molecules were used for the production of 1 butyrate molecule. An alternative model in which the stoichiometry of the interconversion between propionate and butyrate was changed gave similar results, indicating that our conclusions do not depend critically on this assumption (Text S1). This was not surprising because there was hardly any interconversion flux between propionate and butyrate. We solved the full set of flux balance equations for the *in vivo* SCFA production, interconversion and uptake fluxes with the Generalized Reduced Gradient (GRG) Nonlinear Solver algorithm [29]. A full description of the model and computational analysis is given in Text S1.

### *In vivo* bacterial short-chain fatty acid production and host fluxes

The above described model-based method to measure *in vivo* fluxes and SCFA interconversion was applied to the different groups of mice treated with guar gum and to the control group for comparison (Figure 3C). The production and uptake fluxes of acetate were highest, followed by the propionate and butyrate fluxes. This corresponded with the order of SCFA concentrations (Figure 1K). The production flux of acetate was higher than its uptake flux, since 7.1% of the acetate was converted into butyrate (Figure 3C and Table 2), revealing microbial cross-feeding. The production and uptake fluxes of propionate were almost identical, since there was little conversion of propionate into the other SCFAs and back (Figure 3C and Table 2). Interestingly, approximately 50% of butyrate was not produced directly from fibers but rather via dimerization of acetate (Figure 3C and Table 2).

**Table 2:** SCFA interconversion fluxes (mmol/kg/h) for the different Guar Gum groups.

	Guar Gum content (%)			
	0	5	7.5	10
Acetate → Butyrate	0.11±0.01	0.14±0.01	0.32±0.02	0.47±0.02
Butyrate → Acetate	0.00±0.00	0.00±0.00	0.00±0.01	0.00±0.02
Propionate → Acetate	0.00±0.00	0.00±0.01	0.00±0.06	0.00±0.01
Acetate → Propionate	0.00±0.01	0.00±0.01	0.00±0.04	0.00±0.05
Propionate → Butyrate	0.00±0.00	0.00±0.00	0.00±0.00	0.00±0.00
Butyrate → Propionate	0.00±0.00	0.00±0.00	0.00±0.00	0.00±0.01

Data represent means ± SEM for n = 7–8.

The guar gum treatment increased all the fluxes of SCFA production and uptake by the host in a dose-dependent manner (Figure 3C), with the highest fluxes at the highest fiber dose. The relative increase was highest for the propionate production and uptake fluxes (787% increase at 10% guar gum vs. control; p<0.001 and 761% increase at 10% guar gum vs. control; p<0.001, respectively) followed by the acetate production and uptake fluxes (514% increase at 10% guar gum vs. control; p<0.001 and 518% increase at 10% guar gum vs. control; p<0.001, respectively) and the butyrate production and uptake fluxes (273% increase at 10% guar gum vs. control; p<0.001 and 461% increase at 10% guar gum vs. control; p<0.001, respectively).

### Changes in metabolic syndrome markers were inversely correlated with *in vivo* short-chain fatty acid fluxes

**Figure 4:**
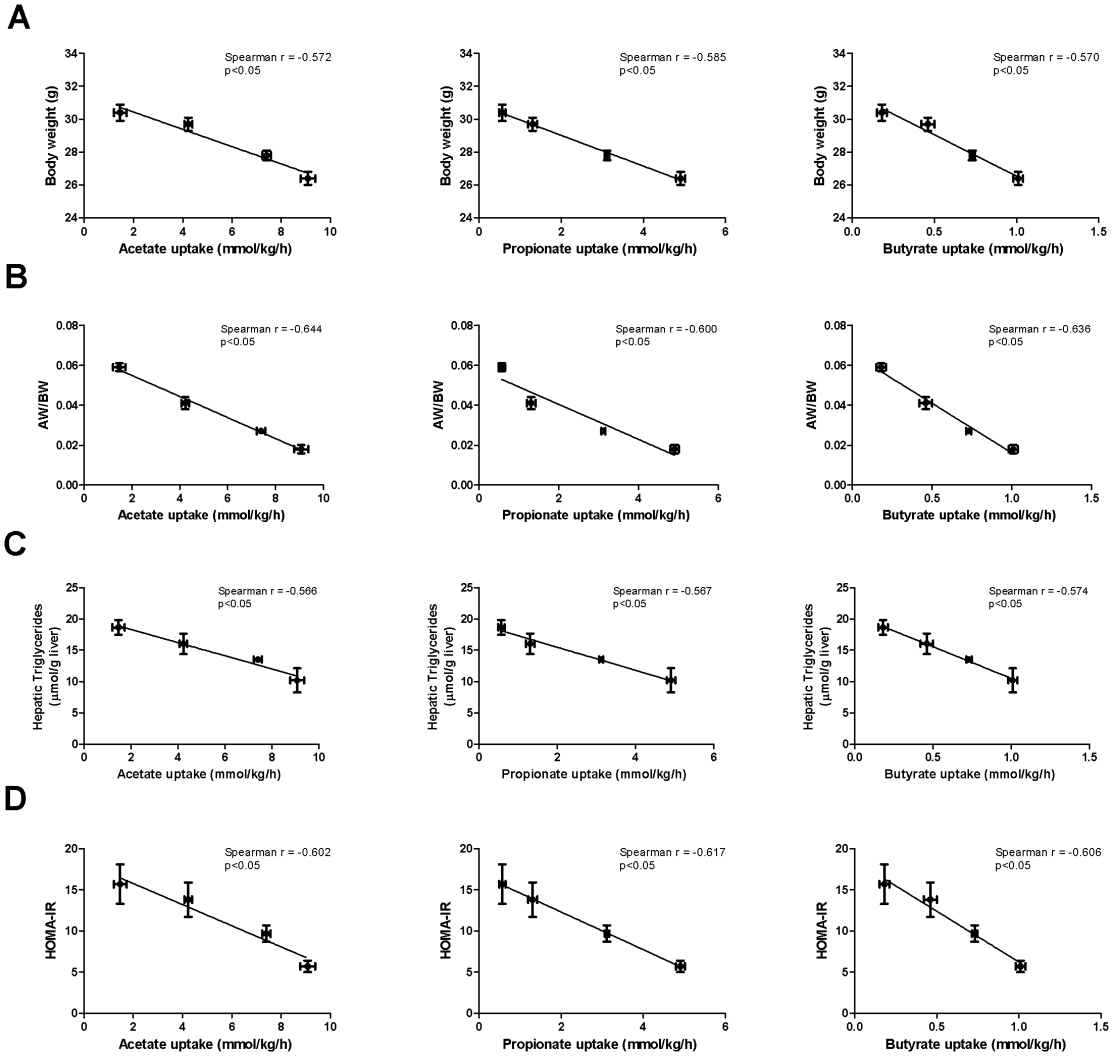
*In vivo* SCFA uptake fluxes correlate inversely with metabolic syndrome markers. Correlation of acetate, propionate and butyrate host uptake fluxes with body weight (A), AW/BW (B), hepatic triglycerides (C) and HOMA-IR (D). The Spearman's correlation coefficient was calculated and the significance level was set at p<0.05.

None of the *in vivo* SCFA fluxes correlated with the cecal SCFA concentrations or expression of the cecal SCFA transporters (p>0.05; Figures S2A and S3A and Tables S2 and S3, respectively). However, *in vivo* SCFA host uptake fluxes did significantly and inversely correlate with body weight, adipose weight, triglycerides, fasted glucose and insulin levels, and HOMA-IR (p<0.05; Figures 4 and S2). In concert, *in vivo* SCFA host uptake fluxes significantly correlated with genes involved in glycolysis and inversely with genes involved in gluconeogenesis and fatty acid synthesis (Table S2). No correlation was found for fatty acid oxidation genes (Table S2). Similar results were found for the *in vivo* SCFA production fluxes (Figure S3 and Table S3). The correlation was better for the uptake fluxes by the host than for the microbial production fluxes, however. The difference between uptake and production fluxes was due to microbial interconversion. The correlation was generally increasing from acetate to butyrate to propionate. Altogether, these data suggest that *in vivo* SCFA fluxes, and not concentrations, are key to understand the beneficial effects of fibers on metabolic syndrome markers.

## Discussion

In this paper we demonstrate that the rate of uptake of SCFAs directly correlates with amelioration of symptoms clustered in the metabolic syndrome. Beneficial effects of SCFAs have been frequently suggested in the literature, but evidence for this contention has been lacking since physiological effects did not correlate with luminal SCFA concentrations. By determining the *in vivo* SCFA fluxes we show for the first time that *in vivo* SCFA fluxes rather than concentrations correlate in an inverse manner with biomarkers of the metabolic syndrome. Here we will discuss how this adds to the evidence for a *causal* relation between fiber intake, SCFA fluxes and attenuation of metabolic syndrome markers.

Human randomized controlled clinical trials showed that guar gum supplementation decreased body weight and fasting plasma glucose and insulin concentrations in both healthy and metabolic syndrome patients [5], [30], [31]. Here, we show that increasing the content of guar gum in a high-fat diet resulted in a dose-dependent decrease in body weight, adipose weight, plasma and hepatic triglycerides, fasting plasma glucose and insulin concentrations and HOMA-IR, together indicating an improvement of the metabolic syndrome. To date, no molecular mechanism for these beneficial effects of guar gum has been described. Two lines of evidence suggest that SCFAs exert a key role in this beneficial effect. First, SCFAs are the main end products of microbial fermentation of dietary fibers in the intestine [6]. Second, SCFA supplementation in the diet protects against dietary-induced obesity and insulin resistance [9], [32], suggesting that microbial SCFAs might do the same. The question then arises how SCFAs might mediate the observed dose-dependent effect of dietary fibers whilst their concentrations do not correlate with the fiber dose and metabolic syndrome markers [17], [19], [20], [33]. It is well known that SCFAs regulate the physiology of the host not only via direct effects in the colon, but also via other organs in the host, such as liver and adipose tissue [6]. Indeed, we showed that guar gum supplementation decreased hepatic triglycerides and expression of hepatic genes involved in fatty acid synthesis, indicating a decreased hepatic lipogenesis [34]. In addition, the decreased lipogenesis parameters were inversely correlated with the *in vivo* SCFA uptake fluxes by the host. Together with the fact that dietary supplementation with SCFAs has been shown to activate AMP-activated protein kinase and subsequently decrease lipogenesis in the liver [6], [9], [35], [36], this suggests that the SCFAs that are taken up by the host are responsible for the decrease in hepatic triglycerides. Next to fatty acid metabolism, we demonstrated that guar gum improved glucose handling, via increased insulin sensitivity. The latter was mediated by peripheral tissues, most likely peripheral muscle and adipose tissue. This is in agreement with the finding that dietary fibers and SCFAs increased oxidative metabolism and insulin sensitivity in muscle and adipose tissue [9], [37]–[39].

While our data suggest that the physiological effect of guar gum is mediated via the uptake fluxes of SCFA into the host, we do not explain what causes the dose-dependent SCFA uptake fluxes in the first place. In general, the uptake rate of a metabolite depends on its own concentration as well as on the capacity and kinetics of its transporter. At steady state, neither the concentration of the SCFAs nor the mRNA expression of the SCFA transporters MCT-1 and SMCT-1 correlated with the SCFA flux. Cecum content and thereby uptake surface did not correlate with the fluxes either. We have not measured transporter kinetics, which leaves the possibility that SCFA transport is regulated at the protein level. Even then, the question remains how SCFAs affect the transporter capacity at protein level, if SCFA concentrations are constant. We note that all our measurements were done after 6 weeks on a high-fat diet, and we do not know how the organism evolved towards the measured state. Possibly the SCFA concentrations underwent dose-dependent dynamics during the 6 weeks in which they adapted to a new steady state through a strong feedback control, a phenomena known as “perfect adaptation” [40], [41]. With a p*K*a of ∼4.8 and a luminal pH around 6.0 (pH 5.5–6.5) the major part of SCFAs is present in the dissociated form [42]. The protons that dissociate from the SCFAs are buffered by an unknown type of transporters that couples the import of SCFA anions to bicarbonate secretion into the intestinal lumen [6]. By modifying the buffer capacity it is thereby possible to increase the uptake of SCFAs and achieve “perfect adaptation”. An alternative possibility, which we cannot exclude, is that the concentration of another metabolite co-varies with the uptake fluxes and takes part in the actual causal mechanism. Next to SCFAs, the gut microbiota produces many metabolites which are involved in the regulation of multiple host metabolic pathways [43]. However, obvious candidates such as lactate were hardly detectable (data not shown).

In conclusion, our data clearly showed that *in vivo* SCFA fluxes and not SCFA concentrations are inversely correlated to metabolic syndrome markers. Together with the known causal effect of increased SCFA in the diet, this provides strong evidence for a causal relation between SCFA uptake flux and metabolic syndrome. Further research should elucidate the role of additional molecular factors that mediate this effect, as well as the mechanism explaining the dose-dependency of the uptake fluxes.

## Supporting Information

Figure S1 Correlation of cecal concentrations of acetate, propionate and butyrate with plasma triglycerides (A), plasma glucose (B) and plasma insulin levels (C). The Spearman's correlation coefficient was calculated and the significance level was set at p<0.05.
(TIF)

Figure S2 Correlation of acetate, propionate and butyrate host uptake fluxes with cecal concentrations of acetate, propionate and butyrate, respectively (A). Correlation of acetate, propionate and butyrate host uptake fluxes with plasma triglycerides (B), plasma glucose (C) and plasma insulin levels (D). The Spearman's correlation coefficient was calculated and the significance level was set at p<0.05.
(TIF)

Figure S3 Correlation of bacterial production fluxes of acetate, propionate and butyrate with cecal SCFA concentrations (A), body weight (B), adipose body weight ratio (C), plasma triglycerides (D), hepatic triglycerides (E), plasma glucose (F) and plasma insulin levels (G) and HOMA-IR (H). The Spearman's correlation coefficient was calculated and the significance level was set at p<0.05.
(TIF)

Table S1 Correlation of cecal acetate, propionate and butyrate concentration with genes involved in SCFA transport, gluconeogenesis, glycolysis, fatty acid synthesis and fatty acid oxidation. The Spearman's correlation coefficient was calculated and the significance level was set at p<0.05.
(DOCX)

Table S2 Correlation of acetate, propionate and butyrate host uptake fluxes with genes involved in SCFA transport, gluconeogenesis, glycolysis, fatty acid synthesis and fatty acid oxidation. The Spearman's correlation coefficient was calculated and the significance level was set at p<0.05.
(DOCX)

Table S3 Correlation of bacterial production fluxes of acetate, propionate and butyrate with genes involved in SCFA transport, gluconeogenesis, glycolysis, fatty acid synthesis and fatty acid oxidation. The Spearman's correlation coefficient was calculated and the significance level was set at p<0.05.
(DOCX)

Text S1 Model description.
(DOC)
