# Supplementary material for: The Short-Chain Fatty Acid Uptake Fluxes by Mice on a Guar Gum Supplemented Diet Associate with Amelioration of Major Biomarkers of the Metabolic Syndrome
Source: PLoS One. 2014 Sep 9;9(9):e107392. doi: 10.1371/journal.pone.0107392 (PMC4159349; doi:10.1371/journal.pone.0107392)
Supplement: Table S1 — Correlation of cecal acetate, propionate and butyrate concentration with genes involved in SCFA transport, gluconeogenesis, glycolysis, fatty acid synthesis and fatty acid oxidation. The Spearman's correlation coefficient was calculated and the significance level was set at p<0.05. (DOCX) [file pone.0107392.s004.docx]

**Table S1.** Correlation of cecal acetate, propionate and butyrate concentration with genes involved in SCFA transport, gluconeogenesis, glycolysis, fatty acid synthesis and fatty acid oxidation. The Spearman’s correlation coefficient was calculated and the significance level was set at p<0.05.

|  | Cecal concentration | | |
| --- | --- | --- | --- |
|  | Acetate | Propionate | Butyrate |
| *SCFA transport* |  |  |  |
| Mct-1 | p<0.05 r=0.366 | p<0.05 r=0.352 | p<0.05 r=0.339 |
| Smct-1 | p<0.05 r=0.418 | p<0.05 r=0.416 | p<0.05 r=0.425 |
|  |  |  |  |
| *Gluconeogenesis* |  |  |  |
| Pepck | NS | NS | NS |
| G6Pase | NS | NS | NS |
| PC | NS | NS | NS |
|  |  |  |  |
| *Glycolysis* |  |  |  |
| HK | NS | NS | NS |
| PK | NS | NS | NS |
|  |  |  |  |
| *Fatty acid synthesis* |  |  |  |
| Fasn | NS | NS | NS |
| Acc1 | NS | NS | NS |
| Acc2 | NS | NS | NS |
| Elovl6 | NS | NS | NS |
|  |  |  |  |
| *Fatty acid oxidation* |  |  |  |
| Cpt-1a | NS | NS | NS |
| Mcad | NS | NS | NS |
| Lcad | NS | NS | NS |
| Aox | NS | NS | NS |

NS, not significant.
