# Supplementary material for: The Short-Chain Fatty Acid Uptake Fluxes by Mice on a Guar Gum Supplemented Diet Associate with Amelioration of Major Biomarkers of the Metabolic Syndrome
Source: PLoS One. 2014 Sep 9;9(9):e107392. doi: 10.1371/journal.pone.0107392 (PMC4159349; doi:10.1371/journal.pone.0107392)
Supplement: Table S2 — Correlation of acetate, propionate and butyrate host uptake fluxes with genes involved in SCFA transport, gluconeogenesis, glycolysis, fatty acid synthesis and fatty acid oxidation. The Spearman's correlation coefficient was calculated and the significance level was set at p<0.05. (DOCX) [file pone.0107392.s005.docx]

|  | Host uptake flux | | |
| --- | --- | --- | --- |
|  | Acetate | Propionate | Butyrate |
| *SCFA transport* |  |  |  |
| Mct-1 | NS | NS | NS |
| Smct-1 | NS | NS | NS |
|  |  |  |  |
| *Gluconeogenesis* |  |  |  |
| Pepck | p<0.05 r=-0.590 | p<0.05 r=-0.592 | p<0.05 r=-0.581 |
| G6Pase | p<0.05 r=-0.666 | p<0.05 r=-0.630 | p<0.05 r=-0.660 |
| PC | p<0.05 r=-0.449 | p<0.05 r=-0.420 | p<0.05 r=-0.444 |
|  |  |  |  |
| *Glycolysis* |  |  |  |
| HK | p<0.05 r=0.618 | p<0.05 r=0.634 | p<0.05 r=0.629 |
| PK | p<0.05 r=0.523 | p<0.05 r=0.553 | p<0.05 r=0.534 |
|  |  |  |  |
| *Fatty acid synthesis* |  |  |  |
| Fasn | p<0.05 r=-0.558 | p<0.05 r=-0.521 | p<0.05 r=-0.574 |
| Acc1 | p<0.05 r=-0.483 | p<0.05 r=-0.446 | p<0.05 r=-0.473 |
| Acc2 | p<0.05 r=-0.516 | p<0.05 r=-0.538 | p<0.05 r=-0.542 |
| Elovl6 | p<0.05 r=-0.431 | p<0.05 r=-0.411 | p<0.05 r=-0.439 |
|  |  |  |  |
| *Fatty acid oxidation* |  |  |  |
| Cpt-1a | NS | NS | NS |
| Mcad | NS | NS | NS |
| Lcad | NS | NS | NS |
| Aox | NS | NS | NS |

**Table S2.** Correlation of acetate, propionate and butyrate host uptake fluxes with genes involved in SCFA transport, gluconeogenesis, glycolysis, fatty acid synthesis and fatty acid oxidation. The Spearman’s correlation coefficient was calculated and the significance level was set at p<0.05.

NS, not significant.
