# Supplementary material for: The Short-Chain Fatty Acid Uptake Fluxes by Mice on a Guar Gum Supplemented Diet Associate with Amelioration of Major Biomarkers of the Metabolic Syndrome
Source: PLoS One. 2014 Sep 9;9(9):e107392. doi: 10.1371/journal.pone.0107392 (PMC4159349; doi:10.1371/journal.pone.0107392)
Supplement: Table S3 — Correlation of bacterial production fluxes of acetate, propionate and butyrate with genes involved in SCFA transport, gluconeogenesis, glycolysis, fatty acid synthesis and fatty acid oxidation. The Spearman's correlation coefficient was calculated and the significance level was set at p<0.05. (DOCX) [file pone.0107392.s006.docx]

**Table S3.** Correlation of bacterial production fluxes of acetate, propionate and butyrate with genes involved in SCFA transport, gluconeogenesis, glycolysis, fatty acid synthesis and fatty acid oxidation. The Spearman’s correlation coefficient was calculated and the significance level was set at p<0.05.

|  | Bacterial production flux | | |
| --- | --- | --- | --- |
|  | Acetate | Propionate | Butyrate |
| *SCFA transport* |  |  |  |
| Mct-1 | NS | NS | NS |
| Smct-1 | NS | NS | NS |
|  |  |  |  |
| *Gluconeogenesis* |  |  |  |
| Pepck | p<0.05 r=-0.591 | p<0.05 r=-0.591 | p<0.05 r=-0.556 |
| G6Pase | p<0.05 r=-0.663 | p<0.05 r=-0.625 | p<0.05 r=-0.639 |
| PC | p<0.05 r=-0.447 | p<0.05 r=-0.416 | p<0.05 r=-0.455 |
|  |  |  |  |
| *Glycolysis* |  |  |  |
| HK | p<0.05 r=0.628 | p<0.05 r=0.578 | p<0.05 r=0.544 |
| PK | p<0.05 r=0.534 | p<0.05 r=0.554 | p<0.05 r=0.521 |
|  |  |  |  |
| *Fatty acid synthesis* |  |  |  |
| Fasn | p<0.05 r=-0.562 | p<0.05 r=-0.515 | p<0.05 r=-0.572 |
| Acc1 | p<0.05 r=-0.478 | p<0.05 r=-0.442 | p<0.05 r=-0.482 |
| Acc2 | p<0.05 r=-0.531 | p<0.05 r=-0.537 | p<0.05 r=-0.555 |
| Elovl6 | p<0.05 r=-0.434 | p<0.05 r=-0.408 | p<0.05 r=-0.430 |
|  |  |  |  |
| *Fatty acid oxidation* |  |  |  |
| Cpt-1a | NS | NS | NS |
| Mcad | NS | NS | NS |
| Lcad | NS | NS | NS |
| Aox | NS | NS | NS |

NS, not significant.
