# Supplementary material for: The Short-Chain Fatty Acid Uptake Fluxes by Mice on a Guar Gum Supplemented Diet Associate with Amelioration of Major Biomarkers of the Metabolic Syndrome
Source: PLoS One. 2014 Sep 9;9(9):e107392. doi: 10.1371/journal.pone.0107392 (PMC4159349; doi:10.1371/journal.pone.0107392)
Supplement: Text S1 — Model description. (DOC) [file pone.0107392.s007.doc]

**Text S1: Model description**

**Model construction**

We constructed a steady-state model to derive the fluxes of *in vivo* short-chain fatty acid (SCFA) production, interconversion and their uptake by the host from the measured isotope distribution patterns. Based on the reaction scheme in Figure 3B, a set of steady-state equations was constructed for all three SCFA tracer infusions, *i.e.* [1-^13^C]acetate, [2-^13^C]propionate and [2,4-^13^C_2_]butyrate. The steady-state model was solved in Excel, which uses the Generalized Reduced Gradient (GRG) Nonlinear Solver algorithm [1]. The model is based on the following assumptions*. i*) All total-mass fluxes (*v*’s below) are independent of the infusion. This is warranted by the use of tracer amounts of labeled SCFAs and moreover, the label distribution was measured at steady state. This then allowed combining the data from the three infusion experiments into one model to calculate the fluxes. *ii*) Two acetate molecules are used for the production of one butyrate molecule and two acetate molecules are produced from one butyrate molecule as shown previously by others [2,3]. The former is consistent with the detection of double-labeled butyrate in our dataset. Analogously, two propionate molecules are used for the production of one butyrate molecule and two propionate molecules are produced from one butyrate molecule. This assumption is supported by mass isotopologue distribution analysis (MIDA) algorithms as shown in our previous work [4] and again by the detection of double-labeled butyrate upon infusion of single-labeled propionate in this study. One acetate molecule is produced from one propionate molecule and *vice versa* one propionate molecule is produced from one acetate molecule, as indicated previously by others [5,6]. *iii*) All fluxes are considered to be equal or higher than zero and *iv*) each of the isotopologue concentrations of acetate, propionate and butyrate is at steady state.

The fluxes *v*_A,0_, *v*_P,0_ and *v*_B,0_ as depicted in Figure 3B in the main text are defined as the bacterial production fluxes from complex carbohydrates for acetate, propionate and butyrate, respectively. Similarly, the fluxes *v*_0,A_, *v*_0,P_ and *v*_0,B_ are defined as the host uptake fluxes for acetate, propionate and butyrate, respectively. The interconversion fluxes are defined as:

*v_B,A_*: 1 Acetate → ½ Butyrate [1]

*v_A,B_*: 1 Butyrate → 2 Acetate [2]

*v_B,P_*: 1 Propionate → ½ Butyrate [3]

*v_P,B_*: 1 Butyrate → 2 Propionate [4]

*v_P,A_*: 1 Acetate → 1 Propionate [5]

*v_A,P_*: 1 Propionate → 1 Acetate [6]

Detailed reaction mechanisms are not considered and, therefore, chemical balances are not complete. There is steady state with respect to mass and isotope enrichment in Acetate (A), Propionate (P) and Butyrate (B). Therefore the sum of fluxes into and out of each pool is zero. The measured normalized mass isotopologue distribution (MID) for acetate is based on m0/z, m1/z and are represented as a_0_ and a_1_ such that a_0_ + a_1_ = 1. Analogously for propionate the measured MID is based on m0/z, m1/z and are represented by p_0_ and p_1_ with p_0_ + p_1_ = 1 and for butyrate the measured MID is based on m0/z, m1/z and m2/z and are represented by b_0_, b_1_, and b_2_ with b_0_ + b_1_ + b_2_ = 1. The normalized measured MID was corrected for natural abundance of ^13^C by multiple linear repression according to Lee *et al.* [7] to obtain the excess normalized MID, with A_0_ and A_1_ for acetate and A_0_ + A_1_ = 1, P_0_ and P_1_ for propionate and P_0_ + P_1_ =1 and B_0_, B_1_ and B_2_ for butyrate and B_0_ + B_1_ + B_2_ =1. According to the model the following mass balances can be written for acetate, propionate and butyrate during steady state in which *v*_x_ are the fluxes corresponding to Figure 3B in the main text in mmol/kg/h.

Acetate: $v_{\inf\_A}+v_{A,0}+2\cdot v_{A,B}+v_{A,P}-(v_{0,A}+v_{B,A}+v_{P,A})=0$ [7]

Propionate: $v_{\inf\_P}+v_{P,0}+2\cdot v_{P,B}+v_{P,A}-(v_{0,P}+v_{B,P}+v_{A,P})=0$ [8]

Butyrate: $v_{\inf\_B}+v_{B,0}+\frac{1}{2}\cdot v_{B,A}+\frac{1}{2}\cdot v_{B,P}-(v_{0,B}+v_{P,B}+v_{A,B})=0$ [9]

in which *v*inf_A, *v*inf_P and *v*inf_B are the mass inflow rates due to infusion of acetate, propionate and butyrate, respectively.

The mass balances and the normalized excess MID are used to formulate the mass balances for each of the mass isotopomers A_0_, A_1_, P_0_, P_1_, B_0_, B_1_ and B_2_ during the infusions of [1-^13^C]acetate (A0_A, A1_A, P0_A, P1_A, B0_A, B1_A, B2_A), [2-^13^C]propionate (A0_P, A1_P, P0_P, P1_P, B0_P, B1_P, B2_P) and [2,4-^13^C_2_]butyrate (A0_B, A1_B, P0_B, P1_B, B0_B, B1_B, B2_B).

All ordinary differential equations (ODEs) for mass isotopomers below are based on

$\frac{d[X_{i}]}{dt}=\sum_{j} (p_{j}^{X_{i}}\cdot v_{j,production}^{X})-\sum_{j} (p_{j}^{X_{i}}\cdot v_{j,conversion}^{X})=0$ [10]

where X stands for either acetate, propionate or butyrate, i stands for the isotopologue (*i.e.* 0,1,2), j stands for the reaction and p for the probability of X_i_ involved in reaction j.

Based on formula 10 we constructed in total 21 ODEs for the acetate, propionate and butyrate infusions.

[1-^13^C]Acetate infusion

$\frac{d[A_{0\_A}]}{dt}=v_{A,O}+v_{A,P}\cdot P_{0\_A}+2\cdot v_{A,B}\cdot B_{0\_A}+v_{A,B}\cdot B_{1\_A}-(v_{0,A}+v_{B,A}+v_{P,A})\cdot A_{0\_A}=0$ [11]

$\frac{d[A_{1\_A}]}{dt}=v_{\inf\_A}+v_{A,P}\cdot P_{1\_A}+2\cdot v_{A,B}\cdot B_{2\_A}+v_{A,B}\cdot B_{1\_A}-(v_{0,A}+v_{B,A}+v_{P,A})\cdot A_{1\_A}=0$ [12]

$\frac{d[P_{0\_A}]}{dt}=v_{P,O}+v_{P,A}\cdot A_{0\_A}+2\cdot v_{P,B}\cdot B_{0\_A}+v_{P,B}\cdot B_{1\_A}-(v_{0,P}+v_{B,P}+v_{A,P})\cdot P_{0\_A}=0$ [13]

$\frac{d[P_{1\_A}]}{dt}=v_{P,A}\cdot A_{1\_A}+2\cdot v_{P,B}\cdot B_{2\_A}+v_{P,B}\cdot B_{1\_A}-(v_{0,P}+v_{B,P}+v_{A,P})\cdot P_{1\_A}=0$ [14]

$\frac{d[B_{0\_A}]}{dt}=v_{B,O}+\frac{1}{2}\cdot v_{B,A}\cdot(A_{0\_A})^{2}+\frac{1}{2}\cdot v_{B,P}\cdot(P_{0\_A})^{2}-(v_{0,B}+v_{A,B}+v_{P,B})\cdot B_{0\_A}=0$ [15]

$\frac{d[B_{1\_A}]}{dt}=\frac{1}{2}\cdot(2\cdot v_{B,A}\cdot A_{0\_A}\cdot A_{1\_A})+\frac{1}{2}\cdot(2\cdot v_{B,P}\cdot P_{0\_A}\cdot P_{1\_A})-(v_{0,B}+v_{A,B}+v_{P,B})\cdot B_{1\_A}=0$ [16]

$\frac{d[B_{2\_A}]}{dt}=\frac{1}{2}\cdot v_{B,A}\cdot(A_{1\_A})^{2}+\frac{1}{2}\cdot v_{B,P}\cdot(P_{1\_A})^{2}-(v_{0,B}+v_{A,B}+v_{P,B})\cdot B_{2\_A}=0$ [17]

[2-^13^C]Propionate infusion

$\frac{d[A_{0\_P}]}{dt}=v_{A,O}+v_{A,P}\cdot P_{0\_P}+2\cdot v_{A,B}\cdot B_{0\_P}+v_{A,B}\cdot B_{1\_P}-(v_{0,A}+v_{B,A}+v_{P,A})\cdot A_{0\_P}=0$ [18]

$\frac{d[A_{1\_P}]}{dt}=v_{A,P}\cdot P_{1\_P}+2\cdot v_{A,B}\cdot B_{2\_P}+v_{A,B}\cdot B_{1\_P}-(v_{0,A}+v_{B,A}+v_{P,A})\cdot A_{1\_P}=0$ [19]

$\frac{d[P_{0\_P}]}{dt}=v_{P,O}+v_{P,A}\cdot A_{0\_P}+2\cdot v_{P,B}\cdot B_{0\_P}+v_{P,B}\cdot B_{1\_P}-(v_{0,P}+v_{B,P}+v_{A,P})\cdot P_{0\_P}=0$ [20]

$\frac{d[P_{1\_P}]}{dt}=v_{\inf\_P}+v_{P,A}\cdot A_{1\_P}+2\cdot v_{P,B}\cdot B_{2\_P}+v_{P,B}\cdot B_{1\_P}-(v_{0,P}+v_{B,P}+v_{A,P})\cdot P_{1\_P}=0$ [21]

$\frac{d[B_{0\_P}]}{dt}=v_{B,O}+\frac{1}{2}\cdot v_{B,A}\cdot(A_{0\_P})^{2}+\frac{1}{2}\cdot v_{B,P}\cdot(P_{0\_P})^{2}-(v_{0,B}+v_{A,B}+v_{P,B})\cdot B_{0\_P}=0$ [22]

$\frac{d[B_{1\_P}]}{dt}=\frac{1}{2}\cdot(2\cdot v_{B,A}\cdot A_{0\_P}\cdot A_{1\_P})+\frac{1}{2}\cdot(2\cdot v_{B,P}\cdot P_{0\_P}\cdot P_{1\_P})-(v_{0,B}+v_{A,B}+v_{P,B})\cdot B_{1\_P}=0$ [23]

$\frac{d[B_{2\_P}]}{dt}=\frac{1}{2}\cdot v_{B,A}\cdot(A_{1\_P})^{2}+\frac{1}{2}\cdot v_{B,P}\cdot(P_{1\_P})^{2}-(v_{0,B}+v_{A,B}+v_{P,B})\cdot B_{2\_P}=0$ [24]

[2,4-^13^C_2_]Butyrate infusion

$\frac{d[A_{0\_B}]}{dt}=v_{A,O}+v_{A,P}\cdot P_{0\_B}+2\cdot v_{A,B}\cdot B_{0\_B}+v_{A,B}\cdot B_{1\_B}-(v_{0,A}+v_{B,A}+v_{P,A})\cdot A_{0\_B}=0$ [25]

$\frac{d[A_{1\_B}]}{dt}=v_{A,P}\cdot P_{1\_B}+2\cdot v_{A,B}\cdot B_{2\_B}+v_{A,B}\cdot B_{1\_B}-(v_{0,A}+v_{B,A}+v_{P,A})\cdot A_{1\_B}=0$ [26]

$\frac{d[P_{0\_B}]}{dt}=v_{P,O}+v_{P,A}\cdot A_{0\_B}+2\cdot v_{P,B}\cdot B_{0\_B}+v_{P,B}\cdot B_{1\_B}-(v_{0,P}+v_{B,P}+v_{A,P})\cdot P_{0\_B}=0$ [27]

$\frac{d[P_{1\_B}]}{dt}=v_{P,A}\cdot A_{1\_B}+2\cdot v_{P,B}\cdot B_{2\_B}+v_{P,B}\cdot B_{1\_B}-(v_{0,P}+v_{B,P}+v_{A,P})\cdot P_{1\_B}=0$ [28]

$\frac{d[B_{0\_B}]}{dt}=v_{B,O}+\frac{1}{2}\cdot v_{B,A}\cdot(A_{0\_B})^{2}+\frac{1}{2}\cdot v_{B,P}\cdot(P_{0\_B})^{2}-(v_{0,B}+v_{A,B}+v_{P,B})\cdot B_{0\_B}=0$ [29]

$\frac{d[B_{1\_B}]}{dt}=\frac{1}{2}\cdot(2\cdot v_{B,A}\cdot A_{0\_B}\cdot A_{1\_B})+\frac{1}{2}\cdot(2\cdot v_{B,P}\cdot P_{0\_B}\cdot P_{1\_B})-(v_{0,B}+v_{A,B}+v_{P,B})\cdot B_{1\_B}=0$ [30]

$\frac{d[B_{2\_B}]}{dt}=v_{\inf\_B}+\frac{1}{2}\cdot v_{B,A}\cdot(A_{1\_B})^{2}+\frac{1}{2}\cdot v_{B,P}\cdot(P_{1\_B})^{2}-(v_{0,B}+v_{A,B}+v_{P,B})\cdot B_{2\_B}=0$ [31]

**Objective function**

The objective function was defined as $\sum_{i} \left(\frac{d[X_{i}]}{dt}\right)^{2}$ in which $\frac{d[X_{i}]}{dt}$ are all the time derivatives of all isotopic fractions of the three SCFAs and they were summed over the three infusion experiments. This objective function was minimized.

|  | Guar gum content (%) | | | |
| --- | --- | --- | --- | --- |
|  | 0 | 5 | 7.5 | 10 |
| Objective function (mmol/h/kg)^2^ | 3.95E-01 | 3.59E-01 | 3.50E-01 | 3.68E-01 |

**Alternative model**

To check the stoichiometric assumptions we made for the propionate-butyrate conversion, we also constructed an alternative model with different stoichiometry and compared the objective function and the fluxes to the standard model for the different guar gum diets to validate if the correlation with the metabolic markers is still valid.

*Alternative model*

Instead of reactions [3] and [4]

1 Propionate → 1 Butyrate [32]

1 Butyrate → 1 Propionate [33]

| Guar Gum content | 0% | | 5% | | 7.5% | | 10% | |
| --- | --- | --- | --- | --- | --- | --- | --- | --- |
| Model | Standard | Alternative | Standard | Alternative | Standard | Alternative | Standard | Alternative |
| Objective function | 3.95E-01 | 3.95E-01 | 3.59E-01 | 3.86E-01 | 3.50E-01 | 3.59E-01 | 3.68E-01 | 3.68E-01 |
| *v*_A,0_ | 1.54 | 1.54 | 4.20 | 4.20 | 7.09 | 7.10 | 9.46 | 9.45 |
| *v*_A,B_ | 0.00 | 0.00 | 0.00 | 0.00 | 0.00 | 0.00 | 0.00 | 0.00 |
| *v*_A,P_ | 0.00 | 0.00 | 0.00 | 0.00 | 0.00 | 0.00 | 0.00 | 0.00 |
| *v*_0,A_ | 1.47 | 1.59 | 4.23 | 4.23 | 7.40 | 7.41 | 9.08 | 9.07 |
| *v*_P,0_ | 0.54 | 0.60 | 1.21 | 1.26 | 2.97 | 2.97 | 4.73 | 4.73 |
| *v*_P,A_ | 0.00 | 0.00 | 0.00 | 0.00 | 0.00 | 0.00 | 0.00 | 0.00 |
| *v*_P,B_ | 0.00 | 0.00 | 0.00 | 0.00 | 0.00 | 0.00 | 0.00 | 0.00 |
| *v*_0,P_ | 0.57 | 0.66 | 1.31 | 1.38 | 3.12 | 3.12 | 4.91 | 4.91 |
| *v*_B,0_ | 0.11 | 0.11 | 0.16 | 0.16 | 0.31 | 0.31 | 0.41 | 0.42 |
| *v*_B,A_ | 0.11 | 0.03 | 0.14 | 0.14 | 0.32 | 0.06 | 0.47 | 0.47 |
| *v*_B,P_ | 0.00 | 0.00 | 0.00 | 0.00 | 0.00 | 0.00 | 0.00 | 0.00 |
| *v*_0,B_ | 0.18 | 0.18 | 0.46 | 0.46 | 0.73 | 0.73 | 1.01 | 1.01 |

**Statistics**

The principle of Maximum Likelihood Estimation (MLE) is used for the estimation of errors in the parameters. The computed likelihood function L is given by

$\ln(L)=\sum_{i=0-2} (\frac{(\frac{d[A_{i}]}{dt})^{2}}{\sigma_{A_{i}}^{2}}+\frac{(\frac{d[P_{i}]}{dt})^{2}}{\sigma_{P_{i}}^{2}}+\frac{(\frac{d[B_{i}]}{dt})^{2}}{\sigma_{B_{i}}^{2}})$ [34]

This likelihood function is optimized with respect to the different parameters in order to determine their errors [8].

**References**

1. Lasdon LS, Waren AD, Jain A, Ratner M. (1978) Design and testing of a generalized reduced gradient code for nonlinear programming. ACM Trans Math Softw 4: 34-50.

2. Miller TL, Wolin MJ. (1996) Pathways of acetate, propionate, and butyrate formation by the human fecal microbial flora. Appl Environ Microbiol 62: 1589-1592.

3. Pryde SE, Duncan SH, Hold GL, Stewart CS, Flint HJ. (2002) The microbiology of butyrate formation in the human colon. FEMS Microbiol Lett 217: 133-139.

4. den Besten G, Lange K, Havinga R, van Dijk TH, Gerding A, et al. (2013) Gut-derived short-chain fatty acids are vividly assimilated into host carbohydrates and lipids. Am J Physiol Gastrointest Liver Physiol 305: G900-910.

5. Öztürk M. (1991) Conversion of acetate, propionate and butyrate to methane under thermophilic conditions in batch reactors. Water Res 25: 1509-1513.

6. Mucha H, Lingens F, Trösch W. (1988) Conversion of propionate to acetate and methane by syntrophic consortia. Appl Microbiol Biotechnol 27: 581-586.

7. Lee WN, Byerley LO, Bergner EA, Edmond J. (1991) Mass isotopomer analysis: Theoretical and practical considerations. Biol Mass Spectrom 20: 451-458.

8. Lehmann EL, Casella GC. (2003) Theory of point estimation. New York: Springer-Verlag.
